# Blocking P2Y2 purinergic receptor prevents the development of lipopolysaccharide-induced acute respiratory distress syndrome

**DOI:** 10.3389/fimmu.2023.1310098

**Published:** 2023-12-20

**Authors:** Zahra Kargarpour, Sanja Cicko, Thomas C. Köhler, Andreas Zech, Slagjana Stoshikj, Christina Bal, Andreas Renner, Marco Idzko, Ahmed El-Gazzar

**Affiliations:** ^1^ Department of Pulmonology, Medical University of Vienna, Vienna, Austria; ^2^ Department of Pneumology, Medical Center, University of Freiburg, Freiburg, Germany

**Keywords:** lipopolysaccharide, acute respiratory distress syndrome, purinergic receptors, P2RY2, inflammation

## Abstract

Acute respiratory distress syndrome (ARDS) is associated with high morbidity and mortality resulting from a direct or indirect injury of the lung. It is characterized by a rapid alveolar injury, lung inflammation with neutrophil accumulation, elevated permeability of the microvascular-barrier leading to an aggregation of protein-rich fluid in the lungs, followed by impaired oxygenation in the arteries and eventual respiratory failure. Very recently, we have shown an involvement of the Gq-coupled P2Y2 purinergic receptor (P2RY2) in allergic airway inflammation (AAI). In the current study, we aimed to elucidate the contribution of the P2RY2 in lipopolysaccharide (LPS)-induced ARDS mouse model. We found that the expression of *P2ry2* in neutrophils, macrophages and lung tissue from animals with LPS-induced ARDS was strongly upregulated at mRNA level. In addition, ATP-neutralization by apyrase *in vivo* markedly attenuated inflammation and blocking of P2RY2 by non-selective antagonist suramin partially decreased inflammation. This was indicated by a reduction in the number of neutrophils, concentration of proinflammatory cytokines in the BALF, microvascular plasma leakage and reduced features of inflammation in histological analysis of the lung. P2RY2 blocking has also attenuated polymorphonuclear neutrophil (PMN) migration into the interstitium of the lungs in ARDS mouse model. Consistently, treatment of *P2ry2* deficient mice with LPS lead to an amelioration of the inflammatory response showed by reduced number of neutrophils and concentrations of proinflammatory cytokines. In attempts to identify the cell type specific role of P2RY2, a series of experiments with conditional *P2ry2* knockout animals were performed. We observed that *P2ry2* expression in neutrophils, but not in the airway epithelial cells or CD4^+^ cells, was associated with the inflammatory features caused by ARDS. Altogether, our findings imply for the first time that increased endogenous ATP concentration via activation of P2RY2 is related to the pathogenesis of LPS-induced lung inflammation and may represent a potential therapeutic target for the treatment of ARDS and predictably assess new treatments in ARDS.

## 1 Introduction

Acute respiratory distress syndrome (ARDS) is a life-threating disease often linked to lung infection and systemic inflammation, and characterized by rapid disease progression (1, 2). An acute onset of respiratory distress, edema formation and severe hypoxemia associated with high mortality are the most characteristic features of ARDS (3, 4). Despite advances in ventilatory strategies, general care of the critically ill patient (5, 6) and imaging techniques (7) in the past two decades, efficient pharmacotherapy for ARDS has not yet been developed (8). The excessively high rate of failure in drug efficacy in human models underscores the significant need for investigating its underlying mechanisms and subsequently introducing candidate components with the potential to reduce ARDS symptoms (2). Many researchers believe that ARDS is disclosed by a demonstrative inflammatory response (9). Bacteria-derived pneumonia and sepsis are the most common causes of ARDS (10–13). Neutrophilic granulocytes (neutrophils) are the main drivers of the inflammation process, which often proceeds and leads to breakdown of the endothelial barrier and immune-mediated injury of pulmonary tissue (14, 15). The rapid recruitment of neutrophils to the lung vasculature peaks as early as after four hours (16). Accumulation and clustering of neutrophils at sites of tissue injury are classical hallmarks of acute inflammation (17, 18).

Within inflammatory processes or hypoxia, different mechanisms lead to release of nucleotides such as adenosine-5′-triphosphate (ATP), adenosine-5′-diphosphate (ADP), uridine-5′-triphosphate (UTP) or uridine-5′-diphosphate (UDP) into the extracellular space (19–21). These extracellular nucleotides mediate various cellular reactions via the P2 purinergic receptors (22). Extracellular ATP binds to cell-surface P2 purinergic receptors including 8 transmembrane domain, containing P2Y receptors (P2Y1, P2Y2, P2Y4, P2Y6, P2Y8, P2Y11, P2Y12, and P2Y13 isoforms) (23, 24) and the ligand-gated ion-conducting P2X receptors, of which 7 receptor subunits have been described (P2X1–P2X7) (25, 26). P2R activation is linked to a wide range of cellular responses, including cell migration, cytokine secretion, release of reactive oxygen species or apoptosis. Functional P2Rs are expressed on both inflammatory and lung structural cells (20, 27, 28). It is widely known that certain P2R subtypes play a role in the pathogenesis of lung illnesses such as bronchial asthma and chronic obstructive pulmonary disease (28–30). Extracellular ATP levels have been found to be higher in bronchoalveolar lavage (BAL) fluid from ARDS patients (9). However, given the broad expression of purinergic receptors, it is likely that more than one P2R subtype is involved.

Previously, it has been shown that the extracellular purines such as ATP exert an important role during the induction and maintenance of asthmatic inflammatory reactions (31). Furthermore, cell types implicated in the pathophysiology of bacterial infectious diseases of the lung or ARDS development are affected by purinergic signaling in various ways, e.g. chemotaxis and extended survival of neutrophils (32). Hence, upon activation, neutrophils and macrophages express functional P2Y and P2X receptors, which are sensitive to increasing extracellular ATP concentrations and are known to increase the release of proinflammatory cytokines (33). Therefore, the role of extracellular nucleotides and their corresponding receptors in ARDS as well as the pathophysiology of infectious respiratory diseases has been suggested.

In this study, we provide for the first time direct *in vivo* evidence of the contribution of P2RY2 in acute LPS-induced ARDS. We observed that *P2ry2* expression was upregulated both in lung tissue and immune cells in an ARDS mouse model. *P2ry2* expression is critical for the amelioration of ARDS inflammatory effects. Furthermore, neutrophils expressing *P2ry2* are mainly involved in LPS-induced inflammation. Altogether, these data indicated a pivotal role of P2RY2 signaling in hematopoietic cells for the development of ARDS.

## 2 Materials and methods

### 2.1 Mice

C57BL/6 mice (6-8-week-old) wild type, *CCT*-Cre × *P2ry2* ^fl/fl^, *LysM*-Cre × *P2ry2* ^fl/fl^, *CD4*-Cre × *P2ry2* ^fl/fl^ were bred at the animal facilities of the University Hospital Freiburg and Medical University of Vienna. The animal experiments described within this study were approved by the Animal Ethics Committee of the University of Freiburg (Germany) and the Medical University of Vienna (Austria). All mice were maintained in specific pathogen-free (SPF) conditions. Genetically manipulated mice used in this study were described previously (34). To stimulate an LPS-induced acute lung inflammation, anesthetized mice received an intratracheal (i.t.) injection of LPS (Sigma Aldrich, Germany) diluted to a final dose of 300 µg/kg in PBS (Sigma Aldrich, Germany) to a total volume of 50 µL. The applied dosage was based on previous studies (34, 35). Mice were sacrificed 24 hours or 48 hours after LPS exposure depending on the experiment setting. Bone marrow (BM) chimera experiments was performed as described previously (9). Briefly, irradiated wild type or *P2ry2* ^-/-^ recipients (900 cGy) were reconstituted with 5×10^6^ *P2ry2* ^+/+^ or *P2ry2* ^−/−^ BM suspensions intravenously. The following donor/recipient pairs were combined and treated with LPS i.t.: *P2ry2* ^+/+^ BM in *P2ry2* ^+/+^ recipient, *P2ry2* ^+/+^ BM in *P2ry2* ^-/-^ recipient, *P2ry2* ^-/-^ BM in *P2ry2* ^+/+^ recipient, *P2ry2* ^-/-^ BM in *P2ry2* ^-/-^ recipient.

### 2.2 Purinergic receptor antagonist application

The mice received an i.t. administration of either vehicle (1xPBS), 100 µM Suramin (Cayman Chemical, USA), 100 µM PPADS (4-[[4-formyl-5hydroxy-6-methyl-3-[(phosphonooxy)methyl]-2-pyrinidyl]azo]-1,3-benzenedisulfonic acid tetrasodium salt) (Tocris Bioscience, UK) or 4 U/mL apyrase (Sigma Aldrich, Germany) in 80 µL total volume one hour prior (prophylactic) or 24 hours after (therapeutic) LPS instillation. The concentrations were chosen based on established literature (9) and preliminary dose-response studies aiming to achieve effective receptor blockade within our experimental system. Mice were sacrificed 24 hours after both LPS application in the prophylactic and antagonist treatment in the therapeutic setting.

### 2.3 Collection of lung tissue, broncho-alveolar lavage fluid and BALF cells

The animals were killed by i.p. injection of thiopental (200 mg/kg) (WDT veterinary company, Germany) and exsanguinated. BALFs were collected by flushing the lung with one mL of sterile PBS supplemented with 0.1 mM sodium EDTA, three times. The first mL was collected in a separate tube for cytokine measurement using enzyme-linked immunosorbent assay (ELISA). Bronchoalveolar lavage fluid (BALF) was kept on ice until processing. BALF neutrophils and macrophages were isolated using fluorescence activated cell sorting (FACS). Therefore, BALF cells were incubated with anti-Ly-6G (Gr-1) FITC- and anti-F4/80 PE-labeled antibodies in PBS containing 0.5% BSA and 0.01% sodium azide for 20 min after blocking unspecific binding with an unlabeled anti-CD16/32 antibody. The lungs were resected for qPCR analysis. For qPCR, lung pieces were homogenized and stored in Qiazol (Qiagen, Hilden, Germany).

### 2.4 ATP measurements in BALF of mice

The ATP levels were measured in BALF of mice, immediately after collection using ATPlite assay (Perkin Elmer, Waltham, MA) according to the instructions of the manufacturer. The cell lysis step was omitted to avoid any contamination of intracellular ATP (36).

### 2.5 Total cell count and flow cytometry analysis

The BALF was centrifuged (5 min, 1500 rpm), and supernatant of the first mL was stored at -20°C for subsequent analysis of cytokine levels. Cell pellets were resuspended in 200 µL PBS for total cell counting by hemacytometer. The differential cell count analysis was done by flow cytometry (FacsCalibur BD Bioscience; San Diego, CA) as previously described (29). Briefly, mouse BALF cells were incubated with unlabeled rat anti-mouse CD16/CD32 antibody (Mouse BD Fc Block, BD Biosciences, Germany) to block Fc receptors and stained for 20 minutes with APC-conjugated Armenian hamster monoclonal anti-mouse CD11c (Thermo Scientific, Germany), FITC conjugated rat monoclonal anti-mouse Ly-6G (Gr-1) (Thermo Scientific, Germany), PE-Cy7-conjugated rat monoclonal anti-mouse CD3e (Thermo Scientific, Germany), PE-Cy7-conjugated rat monoclonal anti- B220 (Thermo Scientific, Germany) and PE-conjugated monoclonal anti-mouse F4/80 (Miltenyi Biotec, Germany), in PBS containing 0.5% BSA and 0.01% sodium azide. Differential cell counts were analyzed using the software Cellquest version 3.3 (BD Bioscience, San Diego, CA) and FlowJo version 10 (TreeStar Inc., Ashland, OR) (37).

### 2.6 Analysis of cytokine levels

Concentrations of Interleukin-1β (IL-1β), IL-6, macrophage inflammatory protein-2 (MIP-2), keratinocyte-derived chemokine (KC), interferon-γ (IFN-γ) and tumor necrosis factor alpha (TNF-α) were measured in BALF using enzyme-linked immunoassays (ELISA) (R&D Systems, Germany), according to the manufacturer’s instructions. The detection limit was 2 pg/mL. Samples with values below the detection limit were assigned a cytokine concentration of 1 pg/mL.

### 2.7 Histology

As previously described (34) for H&E (Haematoxylin and Eosin) staining, we embedded lung lobes in Tissue-Tek O.C.T. compound (Sakura Fintek, USA) and then froze them in liquid nitrogen. The molds were sectioned into 5 μm slices followed by staining with filtered 0.1% Mayers Hematoxylin (Sigma; MHS-16) and rinsing in cool running ddH_2_O. Next, they were immersed in 0.1% HCl water and then in undiluted Eosin. Subsequently the slides were rinsed in cool running ddH_2_O and dehydrated in 70% EtOH and then in 95% EtOH. Finally, they were placed in xylene and this was repeated in fresh xylene mount and coversliped with Entellan new (Sigma-Aldrich, Germany).

### 2.8 Quantitative PCR analysis of purinergic receptor

The cells were lysed in qiazol to extract total RNA, followed by cDNA synthesis using random-hexamer primers and the First Strand cDNA Synthesis Kit (Thermo-Fisher Scientific GmbH, Germany). Specific primers for various murine purinergic receptors were employed to assess mRNA expression levels. Quantitative PCR utilized Taqman Universal PCR Mastermix (Applied Biosystems, USA), along with pre-formulated primers and probe mixes (Applied Biosystems, USA). PCR was conducted under the following conditions: 2 minutes at 50°C, 10 minutes at 95°C, followed by 45 cycles of 95°C for 15 seconds and 60°C for 1 minute using a thermal cycler (iCycler,Bio-Rad, USA). Glyceraldehyde-3-phosphate dehydrogenase (*Gapdh*) served as the reference gene. Primer sequences are shown in **Supplementary Table 1**.

### 2.9 Plasma leakage assay

Plasma vascular leakage was examined as previously described (38). Briefly, Evans blue dye conjugated to albumin (EBA, Sigma Aldrich, Germany) (20 mg/kg) was injected into the tail vein of mice. 30 minutes later the mice were sacrificed and the lungs were perfused with PBS supplemented with 5 mM EDTA. The perfused lungs were excised en bloc, dried, weighed and snap frozen in liquid nitrogen. The whole lung was homogenized in PBS (1 mL/100 µg tissue) prior to incubation in formamide at 60°C for 18 hours. The optical density of the supernatant was determined spectrophotometrically at 620 nm after centrifugation at 5,000 x g for 30 minutes. The concentration of the extravasated Evans blue in lung homogenate was calculated against the standard curve and the results expressed as µg of Evans blue dye per gram of lung tissue (38).

### 2.10 Polymorphonuclear neutrophil migration into the interstitium and BALF

At different time points after LPS exposure and treatment with suramin or PPADS, the mice were injected intravenously with Alexa 633-labeled rat anti-mouse GR-1 (10 µg) antibody and allowed to circulate for 5 minutes to bind intravascular PMN. After 5 minutes, the mice were killed. After performing BAL, total lung tissue was harvested. The lungs were minced and digested with 125 U/mL collagenase type XI, 60 U/mL hyaluronidase type I-s, and 60 U/mL DNase (all Sigma Aldrich, Germany) at 37°C for 30 minutes. The digested lungs were obtained by being passed through a cell strainer and the cell suspension was centrifuged for 10 minutes at 300 g. The pellet was lysed with red blood-lysis buffer (ammonium chloride lysis buffer, 150 mM NH_4_Cl, 10 mM KHCO_3_, 100 nM EDTA, dH_2_O, pH=7.4) to remove erythrocytes and centrifuged again. The pellet was resuspended in buffer and the cells were counted with a hemocytometer (39).

### 2.11 Statistical analysis

Data are expressed as mean ± SEM and were tested for differences using one-way ANOVA, followed by Bonferroni post-test to correct for multiple comparison with the LPS/vehicle group as reference. Where appropriate, data were analyzed using unpaired t-test for comparisons of data collected from two different treatment groups. For statistical analysis we used GraphPad Prism version 8 (GraphPad Software, USA). Differences were considered significant at *P* < 0.05 and highly significant at P < 0.005.

## 3 Results

### 3.1 ATP-neutralization or blocking of P2RY2 attenuates LPS-induced lung inflammation

**Figure 1:**
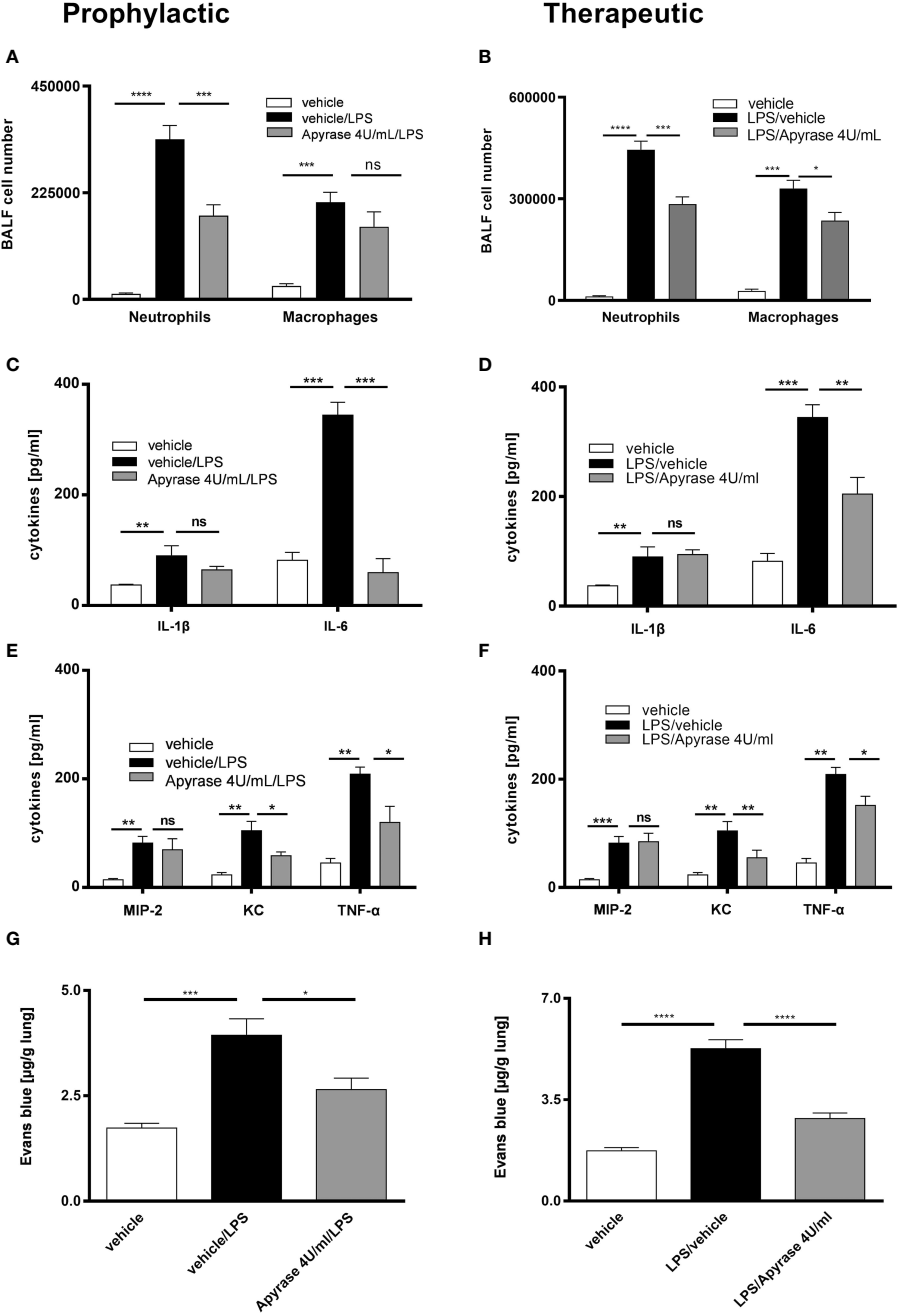
The effect of ATP neutralization by apyrase on LPS-induced ARDS. Mice were treated with 4U/mL apyrase in both prophylactic (left) and therapeutic (right) regimes. **(A, B)** The BALF cells differential count was measured by flow cytometry with both treatment methods. **(C–F)** Concentrations of cytokines IL-1β, IL-6, KC, MIP-2 and TNF-α in BALF were measured by ELISA in both regimes. **(G, H)** Plasma leakage was evaluated spectrophotometrically 30 minutes after Evans blue dye albumin (20 mg/kg), which was injected into the tail vein. Values are given as mean ± SEM. n = 4 mice in each group. *p < 0.05, **p < 0.01, ***p < 0.001, ****p < 0.0001 obtained by comparing each group versus LPS (vehicle/LPS in prophylactic or LPS/vehicle in therapeutic regimes). ns means not significant.

**Figure 2:**
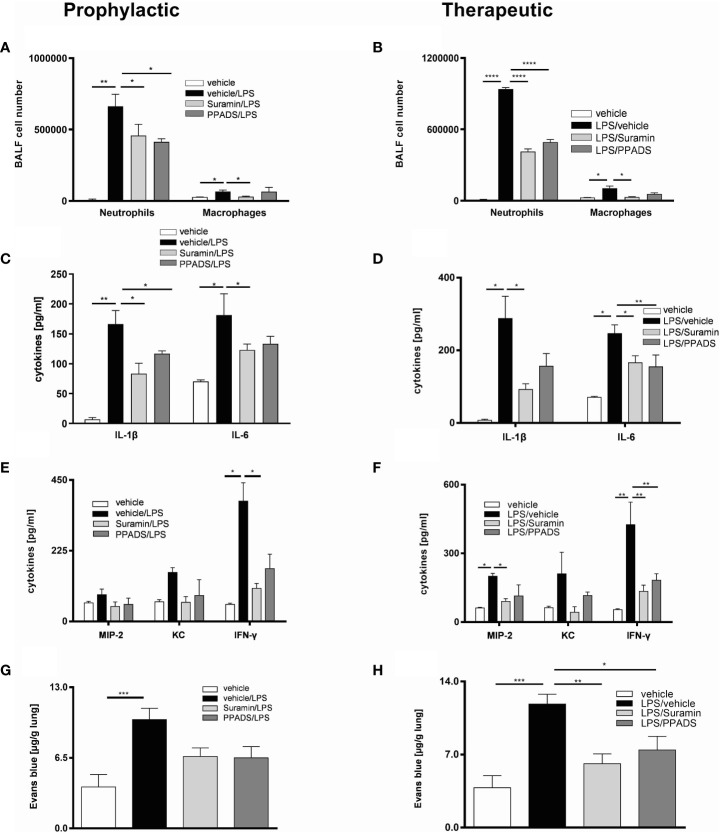
The effect of blocking purinergic receptors on LPS-induced inflammation in mice lungs. The mice were treated with suramin or PPADS in both prophylactic (left) and therapeutic (right) regimes. **(A, B)** The BALF cells differential count was measured by flow cytometry with both treatment methods. **(C–F)** Concentrations of cytokines IL-1β, IL-6, KC, MIP-2 and IFN-γ in BALF were measured by ELISA in both regimes. **(G, H)** Plasma leakage was evaluated spectrophotometrically 30 minutes after Evans blue dye albumin (20 mg/kg), which was injected into the tail vein. Values are given as mean ± SEM. n = 5 mice in each group. *p < 0.05, **p < 0.01, ***p < 0.001, **** p < 0.0001 obtained by comparing each group versus LPS (vehicle/LPS in prophylactic or LPS/vehicle in therapeutic regimes).

To investigate the effect of ATP neutralization on the LPS-induced inflammation, we treated the C57BL/6 wild type mice with 4U/mL apyrase i.t. either one hour prior to or 24 hours after LPS exposure -prophylactic and therapeutic regimes, respectively. The diminished ATP BALF levels due to apyrase treatment (**Supplementary Figure 1**) were accompanied by reduced numbers of neutrophils in both regimes and macrophages only in the therapeutic regime (**Figures 1A, B**). We further observed a reduction of inflammatory cytokines in BALF including IL-6, TNF-α and KC in both regimes (**Figures 1C–F**) as well as decreased levels of Evans blue in microvascular plasma leakage to the lung in both regimes compared to vehicle receiving control animals (**Figures 1G, H**). Notably, both approaches of apyrase treatment showed only a partial reducing effect on IL-1β and MIP-2 secretion in the BALFs. To explore whether the observed effect on reducing inflammation was due to reduced P2RY2 activation and to assess the causative link between P2RY2 function and LPS-induced inflammation, suramin (an inhibitor of P2Y and, with a lower affinity, P2X receptors) was administrated i.t. in either a prophylactic or a therapeutic treatment regime. As a positive control and proof of principle, the nonspecific antagonists PPADS (P2R non-selective antagonist) was applied i.t. in both regimes. As expected, PPADS showed reduced concentration of proinflammatory cytokines, IL-6 and IFN-γ (**Figures 2C–F**). We observed a reduced number of neutrophils not only in a therapeutic but also in a prophylactic treatment (**Figures 2A, B**). Moreover, in therapeutic regime we observed reduced concentrations of IL-1β, IL-6, IFN-γ, and MIP-2 in suramin-treated animals (**Figures 2D, F**). Remarkably, in prophylactic regime we observed decreased concentration of IFN-γ in suramin-treated mice (**Figure 2E**). Microvascular plasma leakage within the lung was also reduced in therapeutic regimes with both antagonist treatments (**Figures 2G, H**). We observed that the effects of both antagonists (suramin or PPADS) on cytokines and Evans blue measurements appeared similar and did not demonstrate clear superiority over each other. In line with our data, H&E staining of the lung indicated reduced inflammatory features of LPS-induced ARDS in both regimes (**Supplementary Figure 2**). Altogether, our results show that ATP-neutralization or blocking of P2RY2 attenuates LPS-induced lung inflammation.

### 3.2 Upregulation of P2RY2 on neutrophils, macrophages and lung tissue in LPS-induced ARDS

**Figure 3:**
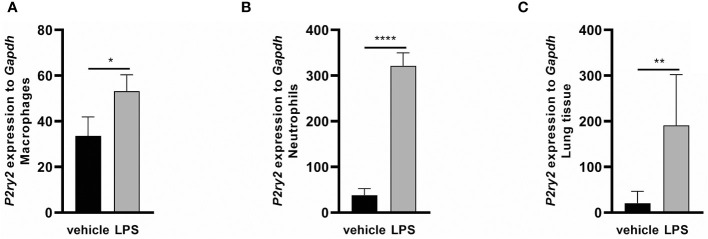
The effect of LPS-induced inflammation on *P2ry2* expression in murine lungs. Animals received vehicle and LPS and 24 hours after LPS i.t. instillation mice exhibited an elevated mRNA expression of *P2ry2* mRNA **(A)** in BALF neutrophils, **(B)** BALF macrophages and **(C)** total lung tissue as determined by qRT-PCR. Statistical analysis was based on unpaired t test. Values are given as mean ± SEM. n = 6 mice in each group. *p < 0.05, **p < 0.01, ****p < 0.0001 fold increase changes are shown.

To evaluate the effect of LPS-induced inflammation on the ATP BALF levels and P2Y2 receptor regulation, C57BL/6 wild type mice were exposed to LPS (300 µg/kg) via i.t. instillation. The measurement of ATP concentration in BALFs after 24 and 48 hours by ATPlite assay showed that LPS administration resulted in increasing ATP concentrations in the BALFs after 24 hours and 48 hours, respectively (**Supplementary Figure 3**). As shown in **Figure 3**, LPS exposure led to an upregulation of *P2ry2* mRNA in BALF macrophages (**Figure 3A**), BALF neutrophils (**Figure 3B**) and total lung tissue (**Figure 3C**) conducted after 24 hours. These data highlight the possible role of P2RY2 in regulating ARDS *in vivo.* The expression of some other family members of purinergic receptors (*P2rx*1, *P2rx4*, *P2rx7*, *P2ry4* and *P2ry6*) was also screened in BALF macrophages and neutrophils after LPS instillation in WT mice. Among them, higher expression of *P2rx4* and *P2ry6* in BALF macrophages and neutrophils was observed (**Supplementary Figure 4**).

### 3.3 *P2ry2* expression has a pivotal role in hematopoietic cells in ARDS

**Figure 4:**
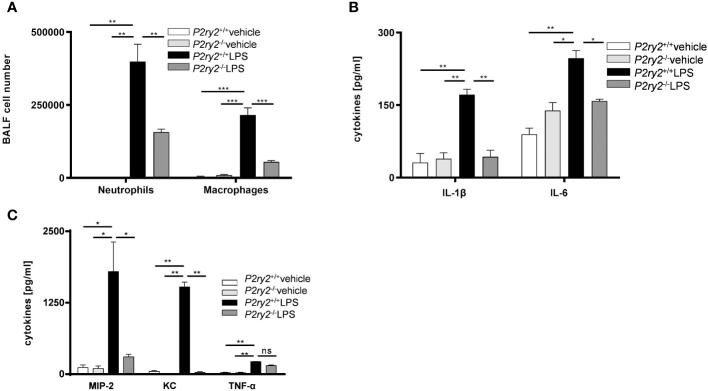
Screening the effect of lacking P2RY2 on LPS-induced ARDS. *P2ry2* knockout mice were treated with either PBS or LPS and the BALFs were collected. **(A)** The number of neutrophils and macrophages in BALFs were measured by flow cytometry. **(B, C)** Concentration of cytokines IL-1β, IL-6, KC, MIP-2 and TNF-α in BALF were measured by ELISA. Statistical analysis was based on Bonferroni post-test. Values are given as mean ± SEM. n = 5 mice in each group. *p < 0.05, **p < 0.01, ***p < 0.001 versus *P2ry2^+/+^* LPS.

**Figure 5:**
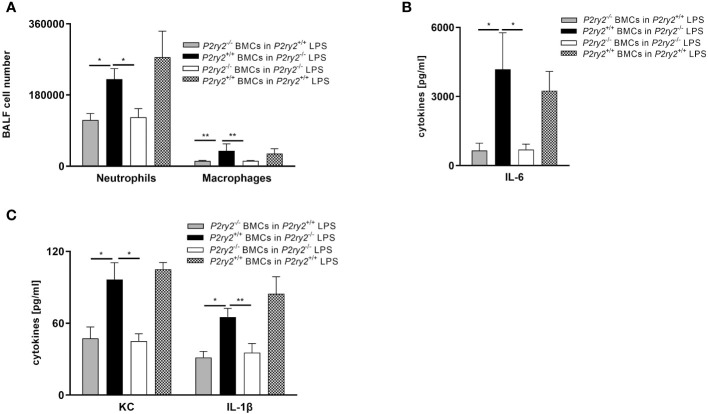
The effect of *P2ry2* expression in murine hematopoietic cells. Different bone marrow chimeric mice were treated with LPS i.t.: Wild type BM in *P2ry2* ^+/+^, Wild type BM in *P2ry2* ^-/-^, *P2ry2* ^-/-^ BM in *P2ry2* ^+/+^, *P2ry2* ^-/-^ BM in *P2ry2* ^-/-^, and then analyzed for lung inflammation. **(A)** The number of neutrophils and macrophages were evaluated in BALFs by flow cytometry. **(B, C)** Concentrations of cytokines IL-6, KC and IL-1β in BALF were measured by ELISA. Statistical analysis was based on Bonferroni post-test. Values are given as mean ± SEM. n = 4-6 mice in each group. *p < 0.05, **p < 0.01 versus *P2ry2* ^+/+^ BMCs in *P2ry2* ^-/-^ LPS.

Next, to provide a conclusive evidence about the contribution of P2RY2 in the pathogenesis of ARDS, we used *P2ry2* knockout mice. *P2ry2* ^-/-^ mice were treated with LPS (300 µg/kg) for 24 h and then we analyzed the immune cells and cytokines in BALF of LPS-challenged mice. We observed that the number of neutrophils and macrophages was significantly decreased in the BALFs obtained from *P2ry2* ^-/-^ mice when compared to wild type mice (**Figure 4A**). We further observed that the concentration of inflammatory cytokines IL-1β, IL-6, MIP-2 and KC was significantly lower in the *P2ry2* ^-/-^ mice compared to wild type (**Figures 4B, C**). Consecutively, to investigate the discrimination between the relevance of *P2ry2* expression in the hematopoietic system and nonhematopoietic system, we performed bone marrow transplantation experiments. We treated different bone marrow chimeric mice with LPS i.t.: *P2ry2* ^+/+^ BM in *P2ry2* ^+/+^ recipient, *P2ry2* ^+/+^ BM in *P2ry2* ^-/-^ recipient, *P2ry2* ^-/-^ BM in *P2ry2* ^+/+^ recipient, *P2ry2* ^-/-^ BM in *P2ry2* ^-/-^ recipient, and then analyzed them for lung inflammation. As shown in **Figure 5**, wild type mice with a lack of *P2ry2* expression in the hematopoietic system (*P2ry2* ^-/-^ BM in *P2ry2* ^+/+^) displayed a significant decrease in lung inflammation, as evidenced by reduced numbers of neutrophils and macrophages in BALF (**Figure 5A**) and decreased concentration of IL-6, TNF-α and MIP-2 in BALFs (**Figures 5B, C**). In contrast, *P2ry2* ^-/-^ animals reconstituted with a wild type hematopoietic system (*P2ry2* ^+/+^ BM in *P2ry2* ^-/-^) showed no protection against LPS-induced lung inflammation. The data indicate that *P2ry2* expression in hematopoietic system has a central role in LPS-induced ARDS inflammatory features.

### 3.4 Cell-type specific *P2ry2* knockout in non-hematopoietic cells resulted no changes in lung inflammation after LPS exposure

**Figure 6:**
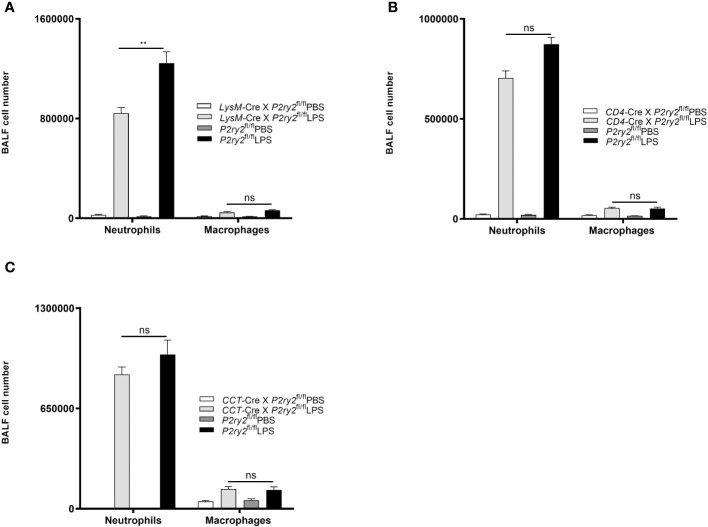
The effect of *P2ry2* conditional knockout mice in different cell types. The experiments were followed by conditional *P2ry2* ^fl/fl^ mice respectively crossed with *LysM*-Cre animals (macrophages/neutrophils), *CD4*-Cre animals (lymphocytes) and *CCT*-Cre animals (airway epithelial cells). All conditional mice were treated i.t. with either LPS or PBS for 24 hours. **(A)** Number of neutrophils and macrophages in *P2ry2* conditional knockout mice in **(A)** macrophages/neutrophils, **(B)** lymphocytes and **(C)** airway epithelial cells. Statistical analysis was based on Bonferroni post-test. Values are given as mean ± SEM. n = 5 mice in each group. **p < 0.01 obtained by comparing LPS treated groups. ns means not significant.

To further characterize the cell-specific influence of P2RY2 function in the pathogenesis of ARDS, we conducted the experiments with conditional *P2ry2* ^fl/fl^ mice crossed with *LysM*-Cre animals (macrophages/neutrophils), CD4-Cre animals (CD4^+^ cells) and CCT-Cre animals (airway epithelial cells). All conditional mice were treated i.t. with LPS for 24 hours. Subsequently, the mice were euthanized and the BALF was collected. In treated animals no protective properties with regard to the LPS-induced lung inflammation was observed. However, there was a significant reduction of neutrophils in the BALF in the *LysM*-Cre × *P2ry2* ^fl/fl^ animals but not the same effect in *CD4*-Cre × *P2ry2* ^fl/fl^ and *CCT-*Cre × *P2ry6* ^fl/fl^ (**Figures 6A–C**). Altogether, these data suggest that the P2RY2 in non-hematopoietic system is not involved in LPS-induced airway inflammation.

### 3.5 Blocking P2 receptors in mice reduce PMN migration into the interstitium of the lungs

**Figure 7:**
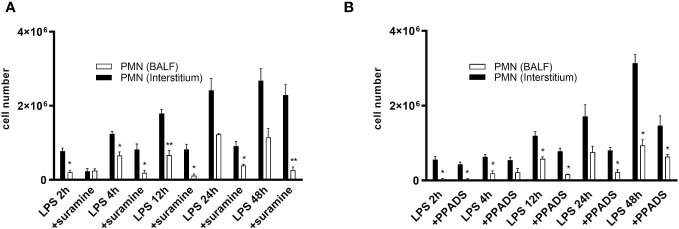
PMN migration into the interstitium and BALF. PMN migration in the various lung compartments (interstitial, alveolar space) was assessed followed by i.t. injection of Alexa 633-labeled rat anti-mouse GR-1 (10 µg) antibody in control C57BL/6 wild type mice and **(A)** Suramin 100 µM- and **(B)** PPADS 100 µM-treated mice over 48 h. Statistical analysis was based on Bonferroni post-test. Values are given as mean ± SEM. n = 4 mice in each group. *p < 0.05, **p < 0.01 versus antagonist LPS.

To describe the impact of P2RY signaling on immune cells during lung inflammation, we evaluated the quantitative movement of polymorphonuclear neutrophils (PMNs) within distinct lung compartments (interstitial and alveolar space) in a spatiotemporal manner. PMN migration was determined in control, PPADS- or suramin-treated mice over 48 hours to detect differences in the time course of PMN trafficking. Following LPS-inhalation, the number of PMNs increased in both lung compartments in a time-dependent manner (**Figures 7A, B**). In the control C57BL/6 wild type mice, LPS-induced PMN accumulation in the interstitium and BALF was significantly higher than in antagonist-treated mice (**Figures 7A, B**). However, there was a greater buildup of PMNs in the interstitium compared to the alveolar space. The results suggest that the suppression of P2RY2 reduces the recruitment of circulating PMNs into the interstitium of the lung and alveolar space in the early stage of lung inflammation. Notably, no significant differences on PMN migration were detected between PPADS- and suramin-treated mice.

## 4 Discussion

In this study, we have established that P2RY2 enhances the typical signs of ARDS disease in an LPS-induced mouse model. Mice treated with apyrase, the ATP-neutralizing enzyme, or PPADS and suramin, non-selective purinergic receptor antagonists, showed a reduction in the proinflammatory cytokine KC concentration, which plays a major role in neutrophil recruitment (40–43). We also observed that apyrase administration had a partial effect on IL-1β and MIP-2 reduction. It is plausible that the interaction between apyrase and inflammatory cells may function as an additional source of IL-1β and MIP-2, in the initial time of the inflammatory response (44). Our data also displayed diminished production of proinflammatory cytokines IL-6 and IFN-γ in LPS-exposed ARDS mice models, challenged with suramin or PPADS. This might be explained by the interplay between IL-6 and IFN-γ signaling which governs neutrophil trafficking and apoptosis during acute inflammation (45). Further, histological analysis of the lung confirmed reduced inflammatory features in the airway of the lung following administration of suramin or PPADS in both prophylactic and therapeutic regimes.

The excessive infiltration of neutrophils into the pulmonary airspace is the main cause for the acute inflammation and lung injury and a common clinical picture of modern intensive care medicine (41). Exposure to LPS intratracheally triggers a hyperinflammatory ARDS phenotype, leading to a substantial recruitment of neutrophils to the lungs, evident through heightened neutrophil levels in the bronchoalveolar lavage fluid (BALF) and increased PMN-trafficking within lung compartments (interstitial, alveolar space) (39, 46). Interestingly, blocking P2R by suramin or PPADS also prevents the development of lung inflammation indicated by reducing PMN influx into the lungs of inflammatory cells of the immune systems. Moreover, we found that i.t. applying of suramin or PPADS, either before or after LPS administration, significantly diminished vascular leakage in the lungs. Given that vascular leakage is a crucial aspect of pulmonary edema in ARDS (47), the significant improvement after antagonist application supports the crucial role of ATP signaling and purinergic receptors in ARDS development. We thereby manifest a functional role for the purinergic receptors in LPS-induced lung inflammation in the mouse lung *in vivo.*

Our findings showing strong upregulation of *P2ry2* at mRNA level in BALF neutrophils, macrophages and lung tissue of the ARDS mouse models suggest that P2RY2 contributes to the accumulation of neutrophils and macrophages in the lung. The mRNA expression does not always directly correspond to protein expression due to various post-transcriptional and translational regulatory mechanisms (48, 49). Therefore, future studies to elucidate the protein expression of the P2RY2 would provide a more comprehensive understanding of its involvement in the observed outcomes. Our observation is in line with the previous findings showing higher expression levels of *P2ry2* in chronic obstructive pulmonary disease (COPD) patients (27) and the role of P2Y2 receptor in chemotaxis of dendritic cells and eosinophils in allergic lung inflammation (50). Additionally, our recent research highlights the involvement of the P2Y2 receptor in the HDM-driven allergic airway inflammation model (34). We found that P2Y2 amplifies the production of proinflammatory cytokines in airway epithelial cells, monocytes, and dendritic cells (34). Furthermore, the P2Y2 receptor facilitates the chemotaxis of dendritic cells and eosinophils during allergic lung inflammation (50).

Our sought definitive experimental proof about the role of P2RY2 in ARDS has been derived from mouse model experiments and a study of *P2ry2*-deficient mice. We demonstrated for the first time that *P2ry2* deficient animals have a reduced pulmonary inflammation after LPS i.t. exposure. We found that the number of neutrophils and macrophages combined with inflammatory cytokines dramatically decreased in *P2ry2^-/-^* mice compared to wild type mice. The role of P2RY2 and its specific impact on neutrophils versus macrophages as a driver of inflammation remains an intriguing area for further exploration. In this context, it has been shown that P2RY2 is critical for neutrophil activation (51) and plays a crucial role in controlling inflammatory gene expression on neutrophils (52). On the other hand, signaling through P2RY2 enhances macrophage IL-1β production (53). Moreover, P2RY2 activation in macrophages has been linked to inflammatory cytokine production such as TNF-α and IL-1β, but direct control of inflammation in macrophages is not well understood (54).

In the following series of experiments with chimera BM *P2ry2* ^-/-^ and wild type animals, it was revealed that expression of *P2ry2* on hematopoietic cells might account for an inflammatory effect of ARDS. *P2ry2* ^-/-^ mice reconstituted with wild type bone marrow did not show a reduction in LPS-induced lung inflammation compared to *P2ry2* ^-/-^ and wild type mice reconstituted with *P2ry2* ^-/-^ bone marrow. To verify the chimera results, we used different cell-type-specific conditional *P2ry2*-deficient mice. Therefore, we specified that neutrophils are mainly involved in LPS-induced inflammation. According to the effective function of CD4 cells in controlling critical aspects of lung immunity (55) and airway epithelial cells as the initial cell type impacted by inhaled components (56) we performed complementary experiments. Conditional knockout mice in non-hematopoietic system including airway epithelial cells and lymphocytes indicated no effect on reducing inflammatory features represented by number of neutrophil and macrophage cells in the BALFs. Our data show for the first time that P2RY2 in the hematopoietic system is important in the pathogenesis of ARDS.

Recent studies have suggested that the peripheral airways play an important role in the pathophysiology of ARDS (57). The mechanism of epithelial injury and denotation are not completely understood but are likely to result from changes in shear stress due to reopening of their collapsed small airways or non-collapsed flooded airways (58, 59). To have a better understanding of the disease mechanism, it is therefore suggested to investigate other members of purinergic receptors family for further studies in the future.

Notably, our current study does not exclude the potential contribution of P2X receptors in ARDS. Indeed, we observed similar effects of non-selective P2Y receptors antagonist suramin (60, 61) or P2 receptors antagonist PPADS (62, 63) on reducing cytokine concentrations and microvascular plasma leakage measurements in the therapeutic regime experiments. In addition, we found that expression of *P2x* receptors including *P2x4* and *P2x7* at mRNA level increased in macrophages of LPS-treated mice. These data are in line with previous reports implying the involvement of P2X receptors in the lung inflammatory response (64, 65). Moreover, previous study manifested the direct involvement of the P2X7 pathway in triggering the inflammatory reaction responsible for ARDS (9).

In summary, the results of the present study show conclusively for the first time the pivotal role of P2RY2 in the pathogenesis of ARDS. These data showed that enhanced endogenous ATP concentration, via activation of purinergic receptors P2RY2 is related to the pathogenesis of LPS-induced lung inflammation in mouse model, and might be a potential therapeutic target for treatment of ARDS patients and predictably evaluate new treatments on organ function in ARDS. This study shed some light on the underlying mechanism and potential components resulting in the ARDS disease.

## Data availability statement

The raw data supporting the conclusions of this article will be made available by the authors, without undue reservation.

## Ethics statement

The animal study was approved by Mice experiments were performed in accordance to the local ethic committee of Freiburg University (Germany) and the Medical University of Vienna (Austria). The study was conducted in accordance with the local legislation and institutional requirements.

## Author contributions

ZK: Data curation, Formal analysis, Methodology, Validation, Writing – original draft. SC: Data curation, Formal analysis, Methodology, Validation, Writing – review & editing. TK: Data curation, Formal analysis, Methodology, Validation, Writing – review & editing. AZ: Writing – review & editing. SS: Data curation, Formal analysis, Writing – review & editing. CB: Data curation, Formal analysis, Writing – review & editing. AR: Data curation, Formal analysis, Writing – review & editing. MI: Conceptualization, Formal analysis, Funding acquisition, Investigation, Resources, Supervision, Validation, Writing – review & editing. AE-G: Conceptualization, Investigation, Project administration, Supervision, Visualization, Writing – original draft, Writing – review & editing.

## Glossary

**Table:**

AAI	Acute airway inflammation
ADP	Adenosine diphosphate
ANOVA	Analysis of variance
ARDS	Acute respiratory distress syndrome
ATP	Adenosine triphosphate
BALF	Broncho-alveolar lavage fluid
BMCs	Bone marrow cells
C57BL/6	C57 black 6
cDNA	complementary deoxyribonucleic acid
COPD	Chronic obstructive pulmonary disease
Cre	Cyclization recombination or causes recombination
DCs	Dendritic cells
ddH2O	Double distilled H_2_O
EDTA	Ethylenediaminetetraacetic acid
ELISA	Enzyme-linked immunoassay
FACS	Fluorescence-activated cell sorting
FITC	Fluorescein isothiocyanate
Fl	flox
GAPDH	Glyceraldehyde-3-phosphate dehydrogenase
H&E	Hematoxylin and eosin
HDM	House dust mite
i.p.	Intraperitoneal
i.t.	Intratracheal
IFN-γ	Interferon gamma
IL	Interleukin
IPF	Idiopathic pulmonary fibrosis
KC	Keratinocytes-derived chemokine
KO	Knockout
LPS	Lipopolysaccharide
Ly6G	Lymphocyte antigen 6 complex locus G6D
MIP-2	Macrophage-inflammatory protein 2
ns	not significant
P2R	Purinergic-type 2-receptor
PBS	Phosphate buffer saline
PE-Cy7	Phycoerythrin-Cyanine7
PMN	Polymorphonuclear neutrophil
PPADS	Pyridoxalphosphate-6-azophenyl-2’,4’-disulfonic acid
qPCR	Quantitative polymerase chain reaction
RNA	Ribonucleic acid
SEM	Standard error of the mean
specific	pathogen-free (SPF)
TaqMan	Taq Polymerase + PacMan
TNF-α	Tumor necrosis factor alpha
TRPV4	Transient receptor potential vanilloid 4
UDP	Uridine diphosphate
UTP	Uridine triphosphate
WT	Wild type
