# Supplementary material for: Blocking P2Y2 purinergic receptor prevents the development of lipopolysaccharide-induced acute respiratory distress syndrome
Source: Front Immunol. 2023 Dec 20;14:1310098. doi: 10.3389/fimmu.2023.1310098 (PMC10765495; doi:10.3389/fimmu.2023.1310098)
Supplement: Supplementary file 1 [file DataSheet_1.docx]

**Supplementary figures:**

**Supplementary Fig. 1:** ATP-neutralization by apyrase attenuates ATP concentrations in the mice BALFs. Animals were sacrificed and the BALFs were then collected. A significant decrease in ATP levels in BALFs is indicated followed by apyrase instillation in both therapeutic prophylactic regimes. Data are means ± SEM, n = 5 , ***p < 0.0005; ****p < 0.0001.

**Supplementary Fig. 2:** Blocking of P2RY2 attenuates LPS-induced lung inflammation. Lung sections of the LPS-treated mice challenged with suramin and PPADS in both regimes were stained with representative H&E for histological examination (magnification×400).

**Supplementary Fig. 3:** Mice treated with LPS had higher ATP levels in their lungs. A significant rise in ATP levels in the BALF followed by exposure to LPS (300 g/kg/i.t.) after 24 and 48 hours compared to controls. Animals were sacrificed and the BALFs were then collected. Data are means ± SEM, n = 5–10, *p < 0.05; **p < 0.01.

**Supplementary Fig. 4:** The effect of LPS-induced inflammation on purinergic receptors expression in murine BALF cells. WT mice received vehicle and LPS and followed by 24 hours after LPS i.t. instillation, expression of P2X1, P2X4 and P2X7 mRNA in (A) BALF neutrophils, (B) BALF macrophages and P2Y4 and P2Y6 in (C) BALF neutrophils, and (D) BALF macrophages was determined by qRT-PCR. Statistical analysis was based on unpaired t-test. Values are given as mean ± SEM. n = 3 mice in each group. *p < 0.05, **p < 0.01 and ***p < 0.001fold increase changes are shown.

**Supplementary table**

**Supplementary** table 1. Taqman primer sequences for quantitative PCR. Sequence direction is from 5ꞌ to 3ꞌ.

| Primer name | Forward | Reverse |
| --- | --- | --- |
| gapdh | TCTCTGCTCCTCCCTGTTCC | GCCAAATCCGTTCACACCGA |
| p2rx1 | TTTCCGTCTGATCCAGTTGGTG | TCACTGACACACTGCTGATAAGG |
| p2rx4 | AACACTTCTCAGCTTGGATTCCG | TTCACGGTGACGATCATGTTGG |
| p2rx7 | GGCATCCGTTTTGACATCCTG | CCAAAGTAGGACAGGGTGGATC |
| p2ry2 | GCATCCTCACCACCTCAAGAG | GTGCCATTGATGGTGCTATTCC |
| p2ry4 | CACATCAGGGGGAACTAAGAGTAC | GTCAATGGTGTTCTGCTGCTTC |
| p2ry6 | CTGGCACTTCCTCCTAAAACATC | CAAGCCTGGAGCCTGGATG |
