# Supplementary figures and images for: Blocking P2Y2 purinergic receptor prevents the development of lipopolysaccharide-induced acute respiratory distress syndrome
Source: Front Immunol. 2023 Dec 20;14:1310098. doi: 10.3389/fimmu.2023.1310098 (PMC10765495; doi:10.3389/fimmu.2023.1310098)

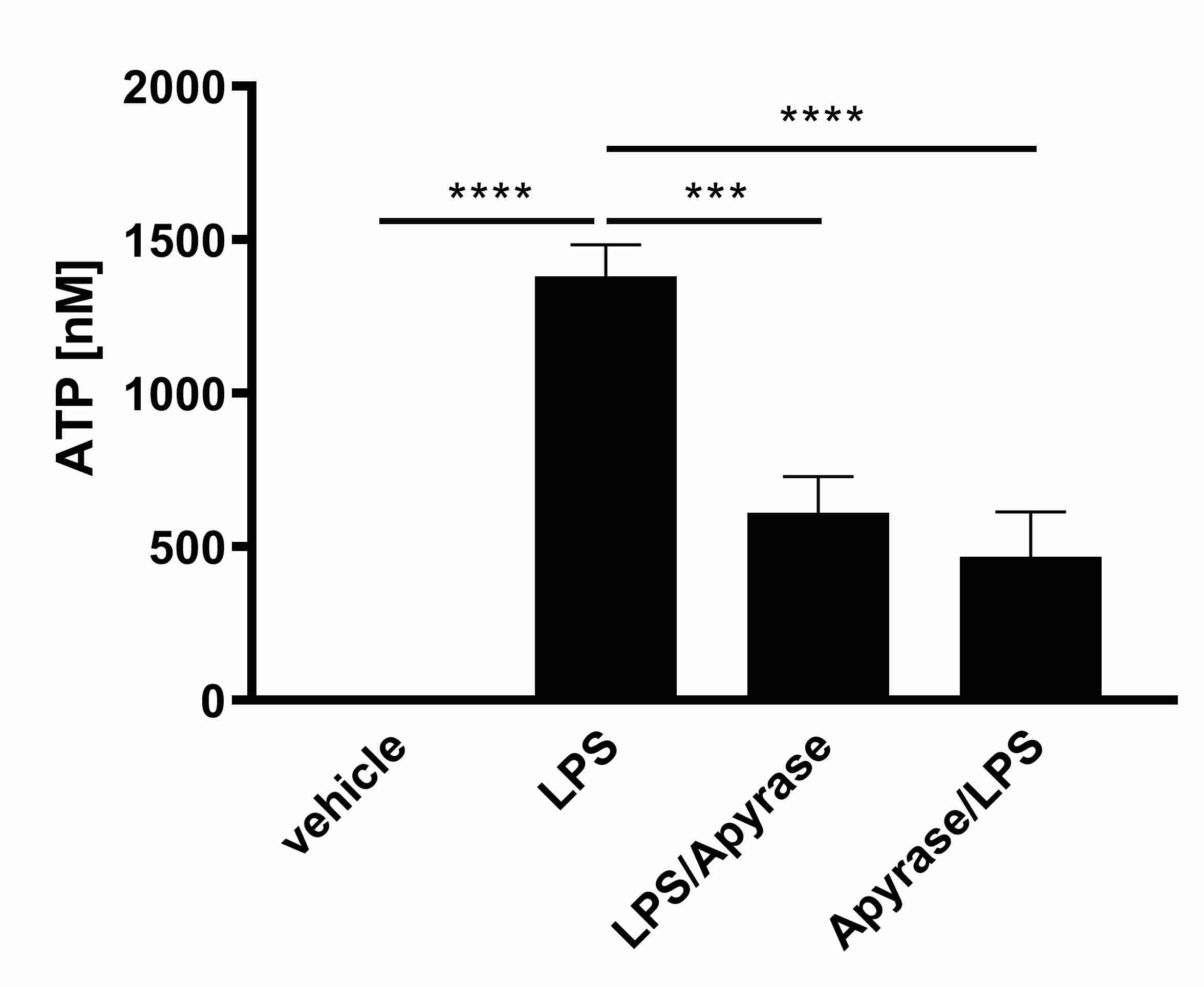

Supplement: Supplementary file 2 [file Image_1.jpeg]

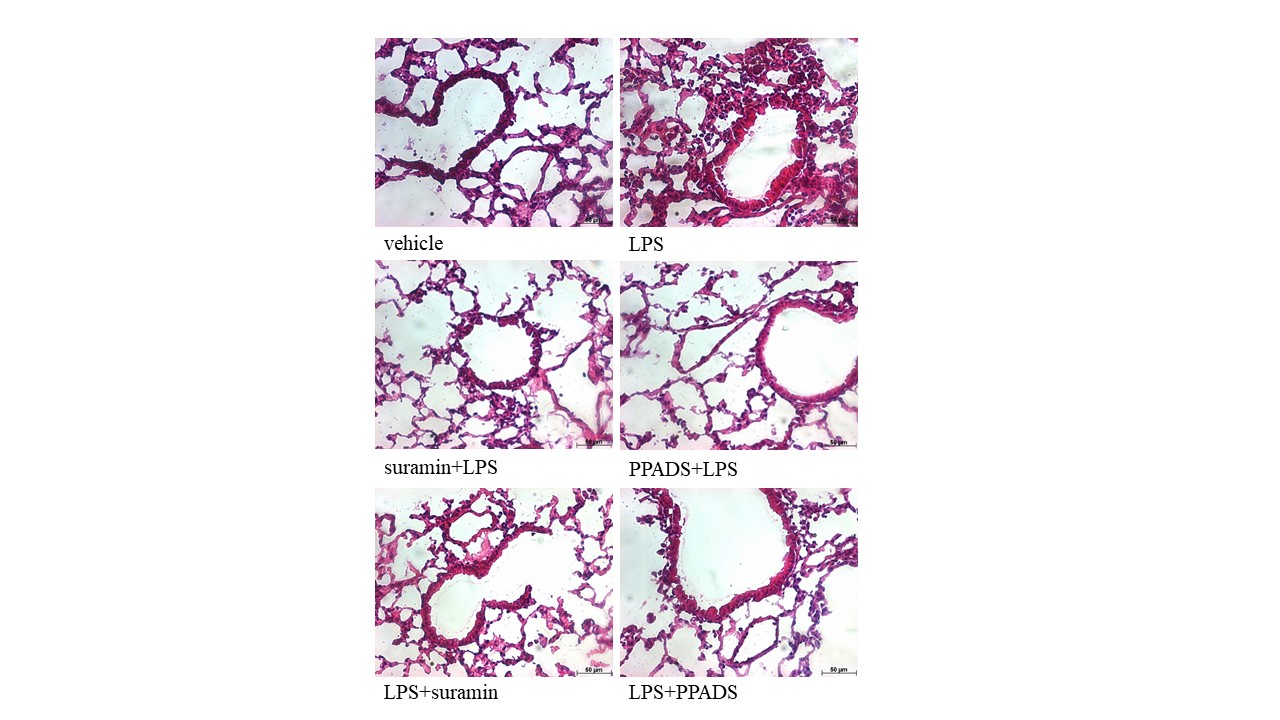

Supplement: Supplementary file 3 [file Image_2.jpeg]

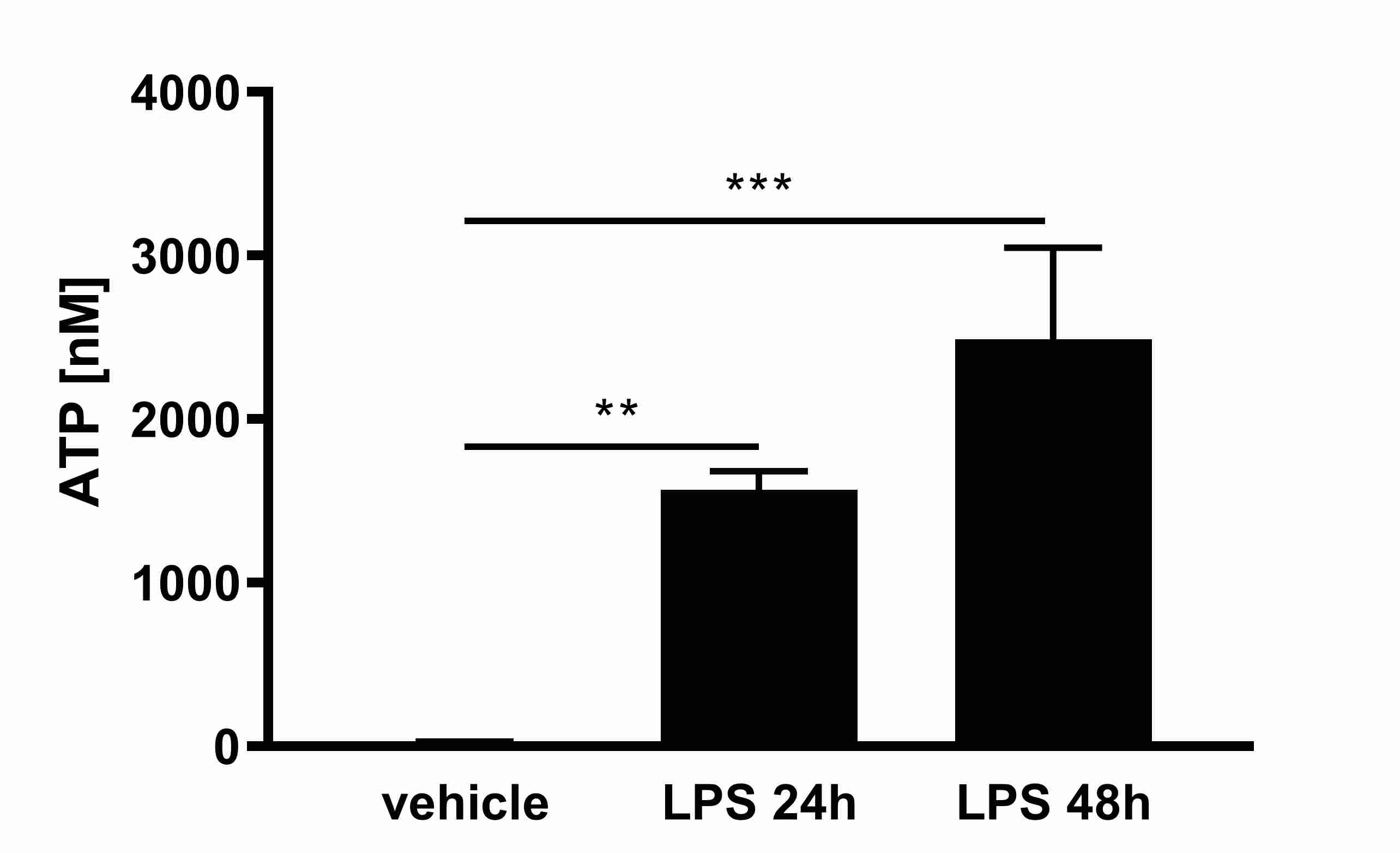

Supplement: Supplementary file 4 [file Image_3.jpeg]

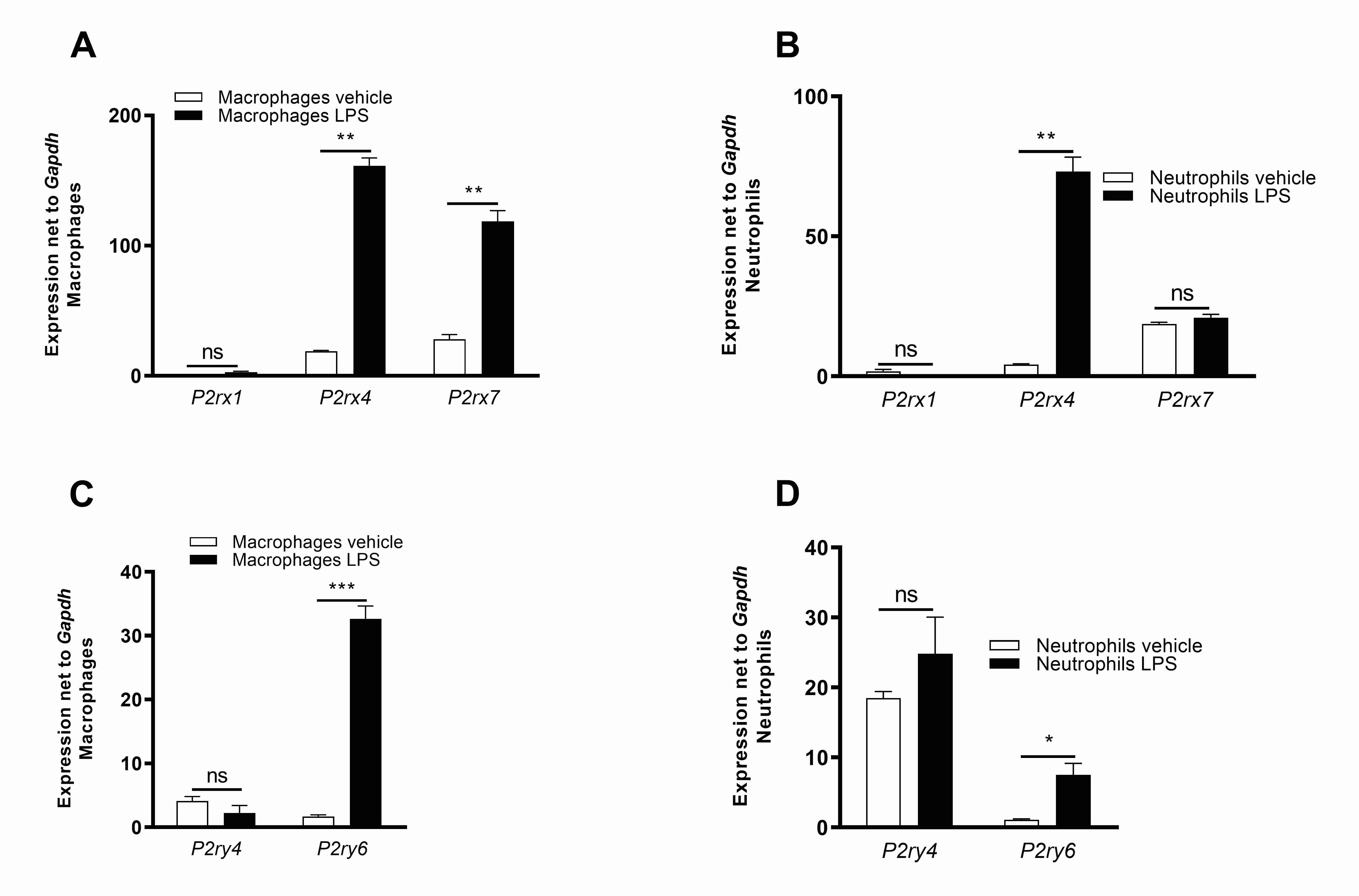

Supplement: Supplementary file 5 [file Image_4.jpg]
